# Photoacoustic Neuroimaging - Perspectives on a Maturing Imaging Technique and its Applications in Neuroscience

**DOI:** 10.3389/fnins.2021.655247

**Published:** 2021-06-10

**Authors:** Silviu-Vasile Bodea, Gil Gregor Westmeyer

**Affiliations:** ^1^Department of Chemistry and School of Medicine, Technical University of Munich (TUM), Munich, Germany; ^2^Institute for Synthetic Biomedicine, Helmholtz Center Munich, Munich, Germany

**Keywords:** optoacoustic imaging, functional brain imaging, molecular contrast agents, calcium and voltage sensors, stroke, brain tumors, image-guided therapy, translational photoacoustic imaging

## Abstract

A prominent goal of neuroscience is to improve our understanding of how brain structure and activity interact to produce perception, emotion, behavior, and cognition. The brain’s network activity is inherently organized in distinct spatiotemporal patterns that span scales from nanometer-sized synapses to meter-long nerve fibers and millisecond intervals between electrical signals to decades of memory storage. There is currently no single imaging method that alone can provide all the relevant information, but intelligent combinations of complementary techniques can be effective. Here, we thus present the latest advances in biomedical and biological engineering on photoacoustic neuroimaging in the context of complementary imaging techniques. A particular focus is placed on recent advances in whole-brain photoacoustic imaging in rodent models and its influential role in bridging the gap between fluorescence microscopy and more non-invasive techniques such as magnetic resonance imaging (MRI). We consider current strategies to address persistent challenges, particularly in developing molecular contrast agents, and conclude with an overview of potential future directions for photoacoustic neuroimaging to provide deeper insights into healthy and pathological brain processes.

## Introduction

Understanding the mechanisms by which the brain gives rise to inner experience, cognition, and behavior remains a central objective of fundamental neuroscience, which will also strongly foster advances in causal therapies of neuropsychiatric diseases.

Mapping spatiotemporal patterns across the vastly different granularities of the brain becomes increasingly important for testing specific circuit models and monitoring pathological processes and therapeutic interventions (Friston, 1994; Sporns et al., 2005; Sporns, 2013).

As a still younger imaging technique, photoacoustic (or synonymously optoacoustic) imaging is entering a crowded field of specialized anatomical and functional imaging modalities. There have been a series of excellent reviews of photoacoustic imaging (PAI) technology which include in-depth comparisons with optical and non-optical imaging techniques in terms of sensitivity to contrast agents, penetration depth, and resolution (Ntziachristos, 2010; Devor et al., 2012; Hu and Wang, 2013; Deán-Ben et al., 2017; Ovsepian et al., 2017; Jeon et al., 2019; Hosseinaee et al., 2020).

This review focuses mainly on photoacoustics’ current capabilities for preclinical brain imaging to extract physiological and molecular contrast and on how the modality can synergize with complementary neuroimaging techniques. We will mostly discuss preclinical research in mouse models used in most current PA neuroimaging studies because the mouse remains the most important organism for modeling human disease, and the dimensions of the mouse brain match well with PA capabilities in terms of tissue penetration and resolution (McGraw et al., 2017). We will then point out emerging opportunities for translation of PA neuroimaging into the clinic.

In structural neuroimaging, electron microscopy (EM) and light-sheet microscopy combined with tissue clearing are systematically improving our maps of brain connections, the so-called connectome (Sporns et al., 2005). Multiphoton microscopy is limited in penetration depth, while computed tomography (CT), magnetic resonance imaging (MRI), and positron emission tomography (PET) offer non-invasive structural characterization of entire brains albeit at lower spatial resolution (Ntziachristos and Razansky, 2010).

*In vivo*, functional and molecular neuroimaging at microscopic scales is dominated by multiphoton techniques, while implantable light guides and miniaturized microscopes illuminate network dynamics in freely moving animals (Malvaut et al., 2020). Widefield epifluorescence, coupled with intrinsic optical imaging (IOS), can cover the entire cortex of small rodents but only superficial layers. Functional MRI (fMRI), functional ultrasound (fUS), and PET can image dynamic processes across the entire mammalian brain at lower spatial and temporal resolutions. For this reason, MRI and PET are probably still the most influential neuroimaging tools, which also connect to neuroscientific studies in humans.

## Imaging Based on the Photoacoustic Effect

The photoacoustic effect relies on the differential thermoelastic expansion of materials after irradiation with non-ionizing electromagnetic waves, causing wideband sound emission (Bell, 1880). Theoretical work into the detection of laser-induced stress waves (Oraevsky et al., 1993, 1997) paved the way for laser-based photoacoustic microscopes (Wada et al., 1986; Cross et al., 1987; Masujima et al., 1988; Maslov et al., 2005) as well as tomographic imaging setups optimized for imaging biological tissue (Kruger et al., 1995; Esenaliev et al., 1996; Oraevsky et al., 1996; Wang et al., 2003b) as detailed in a recent review (Manohar and Razansky, 2016).

Current biomedical PA imaging systems routinely use tunable laser systems in the visible and infrared spectrum. The laser pulses are partially absorbed by tissue chromophores, leading to a conversion into heat with a local temperature increase on the order of millikelvins (Yao and Wang, 2014). The resulting thermoelastic expansion generates a transient pressure increase that propagates through the sample as a broadband acoustic wave which can be recorded by ultrasound detectors and computationally reconstructed to map the distribution of photoabsorbers in the tissue (Ntziachristos, 2010).

The main driver for image contrast is, therefore, differential laser light absorption in biological media. Multispectral laser excitation consequently can localize specific endogenous or exogenous chromophores based on their specific photoacoustic spectra (Razansky et al., 2011).

Because acoustic scattering is orders of magnitude weaker than optical scattering, PA imaging can reach a higher spatial resolution and tissue penetration than traditional, purely optical techniques but still access the rich information contained in endogenous and exogenous photoabsorbing molecules (Ntziachristos and Razansky, 2010).

## Detection Geometries for Photoacoustic Neuroimaging

**FIGURE 1:**
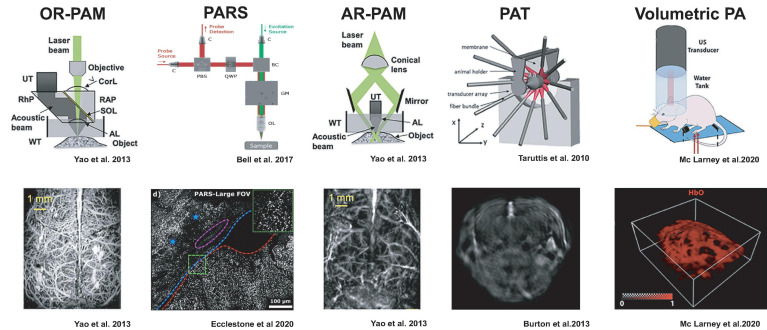
Detection geometries for photoacoustic imaging with example results from neuroimaging. The figures were adapted from the respective references with permission from the authors.

Over the last years, photoacoustic technology has given rise to specialized imaging geometries that find different tradeoffs in spatial and temporal resolution and penetration depth (Deán-Ben et al., 2017). We will briefly summarize the main categories to illustrate practical aspects regarding which samples can be accessed and which results may be expected (Figure 1).

PA microscopy (PAM) can be realized either by restricting the photoacoustic signal generation by focusing a laser beam, so-called optical resolution (OR), or by illuminating the sample more broadly but detecting the ultrasound only from a focal volume, termed acoustic resolution (AR).

OR-PAM relies on tight laser beam focusing onto a selective spot in the tissue and receiving the emitted ultrasound from that optical focal cone on a transducer immersed in a coupling medium. Resolution is limited to the smallest achievable optical focal spot size, and imaging depth is limited to an optical transport mean free path (∼1 mm) (Maslov et al., 2008; Hu et al., 2011b; Hu and Wang, 2013; Yao and Wang, 2013). Thus, similar to optical microscopes, the spatial resolution is diffraction-limited (Deán-Ben et al., 2017; Seeger et al., 2020). OR-PAM necessitates raster scanning across the sample and is often implemented in hybrid microscopes with confocal, multiphoton, or light-sheet microscopy to complement the photoabsorbance maps (Seeger et al., 2020). Since acoustic coupling of the sample with the transducer can be impractical, PA microscopes have been developed which do not require physical coupling between the microscope and sample tissue. Remote sensing photoacoustic microscopy (PARS) is designed to resolve this by co-focusing a continuous probe beam together with the nanosecond excitation beam. The pressure changes from the absorbed light can be detected as changes in the intensity of the reflected probe beam (Bell et al., 2017; Hajireza et al., 2017). This setup may achieve contract-free diffraction-limited resolution at distances of up to 2.5 cm to the imaged tissue (Hajireza et al., 2017) making it suitable for intraoperative applications (Abbasi et al., 2019; Ecclestone et al., 2020).

AR-PAM, in distinction, works with laser excitation that can cover the entire acoustic detection area because the lateral resolution is defined by the acoustic focusing capabilities (Li et al., 2006; Zhang et al., 2006; Park et al., 2014). Image formation occurs from raster scanning of focused transducers and stacking depth profiles from individual laser shots. AR-PAM implementations can reach imaging depths of 5 mm while maintaining 7 μm axial and 30 μm lateral resolutions (Omar et al., 2014). One such implementation, raster-scan optoacoustic mesoscopy (RSOM), can be productively combined with OR-PAM, multiphoton excitation fluorescence, second, and third harmonic generation microscopy (Soliman et al., 2015).

Rather than scanning a single transducer across the specimen, photoacoustic tomography (PAT) uses a multi-element ultrasound detector array to enable the parallel acquisition of an imaging plane through the specimen with a single-shot laser excitation (Kruger et al., 1995; Hoelen et al., 1998; Buehler et al., 2010; Razansky et al., 2011). Light is typically delivered through optical fiber bundles from the side of the transducer array, and tomographic reconstruction of the photoacoustic signals is used to compute the 2D images (Buehler et al., 2010; Lutzweiler and Razansky, 2013). Multiple slices can be acquired by translating either the specimen or the transducer array. By using rapidly tunable pulsed-lasers in visible or near-infrared wavelengths, photoacoustic spectra can be acquired at each subvolume (MSOT), which can allow mapping of the distribution of different photoabsorbers based on characteristic spectra via spectral unmixing methods (Taruttis et al., 2010; Razansky et al., 2011; Tzoumas et al., 2014).

By arranging a two-dimensional array of ultrasonic detectors on a spherical surface, entire image volumes can be reconstructed from single laser pulses without a scanning procedure (Deán-Ben and Razansky, 2013; Xiang et al., 2013; Deán-Ben et al., 2015a). This volumetric PA imaging can also be augmented with multi-spectral information via tunable lasers (Luís Deán-Ben and Razansky, 2014). For practical reasons, laser illumination is usually performed through a central opening of the spherical transducer array, and fluorescence imaging can be added by using coherent fiber bundles (Chen et al., 2017). Current volume rates are limited by the pulse repetition frequency of available laser systems ∼100 Hz but could exceed those of ultrasound. Because of the large volume rates, this scanless image acquisition is very attractive for whole-brain neuroimaging in rodents and other animal models.

## Neuroanatomy of Animal Models

### *Ex vivo* Anatomy

While MR diffusion imaging can deliver useful information on the main fiber tracts in living brains (Jbabdi et al., 2015), *ex vivo* techniques are currently necessary to determine neuronal connectivity near or at synaptic resolution.

Light microscopy is limited in terms of imaging depth in scattering biological media. For this reason, serial mechanical sectioning followed by multiphoton imaging has been used in neuronal tracing (Ragan et al., 2012). *Ex vivo* tissue can also be made optically clear (Chung and Deisseroth, 2013; Renier et al., 2014) and imaged with light-sheet microscopy for non-destructive optical sectioning (Voie and Spelman, 1995; Power and Huisken, 2017; Mano et al., 2018). The resolution in light microscopy can be further improved by tissue expansion (Chen et al., 2015) and stochastic labeling of neurons (Livet et al., 2007).

Neuronal processes can have submicrometer diameters, with dendritic spine necks as small as 0.09 μm in diameter (Bartlett and Banker, 1984; Arellano et al., 2007; Gipson and Olive, 2017) and follow complicated trajectories around each other and relative to the supporting astrocytes, vasculature, and microglia. Therefore, it is challenging to disentangle neuronal processes in densely labeled tissue using, e.g., the brainbow technique (Livet et al., 2007), even at the best-possible optical resolution. Thus, a complete characterization of all synaptic connections will ultimately require imaging the entire brain network via EM. Advances in volumetric EM techniques (Eberle and Zeidler, 2018; Yin et al., 2020) already permitted complete EM brain volumes from *Drosophila* (Zheng et al., 2018) and partial connectomes of larval zebrafish (Hildebrand et al., 2017) to come online, with substantial advances also made for the mammalian cortex (Motta et al., 2019).

PA imaging enables the structural interrogation of the brain at scales ranging from micro- to macroscopic. Either intrinsic or extrinsic photoabsorbers can generate image contrast. The main intrinsic absorbers in brain tissue are DNA, RNA, cytochrome, lipids, and water. Lipid concentrations vary in white matter *vs.* gray matter and between brain regions (Chavko et al., 1993). Since their photoacoustic spectra are masked *in vivo* by hemoglobin, lipid imaging is conducted *ex vivo* after brain perfusion (Li L. et al., 2016). Mesoscale PAT imaging of entire mouse brains *ex vivo* has revealed structures such as the neocortex, cerebellum, cerebral aqueduct, corpus callosum, hypothalamus, hippocampus, inferior colliculus, olfactory bulb, and ventricles (Li L. et al., 2016; Olefir et al., 2019). Furthermore, dopaminergic neurons contain neuromelanin, an endogenous pigment that can be visualized *ex vivo* by photoacoustic tomography (Olefir et al., 2019).

PA microscopy, on the other hand, can resolve DNA/RNA (Yao et al., 2010), lipids (Wang et al., 2011), cytochromes (Zhang et al., 2013), hemoglobin (Yao et al., 2015), and melanin (Zhang et al., 2006) by selecting the appropriate wavelengths of laser illumination. Non-labeled brain tissue blocks measuring a couple of cubic millimeters were serially sectioned, scanned, and volumetrically reconstructed, allowing the mapping of nuclei positions within the blocks (Wong et al., 2017). PA prescans of unstained samples leveraging the intrinsic contrast of the previously mentioned photoabsorbers might provide a useful reference before executing complex fixation or staining protocols, leading to geometric tissue distortion.

Besides imaging the central nervous system, the spinal cord and peripheral nerves can also be accessed by PAI. *Ex vivo* PAT at an excitation wavelength of 1730 nm was employed to assess white-matter loss after spinal cord injury in adult rats (Wu et al., 2014). Myelin is the principal lipid constituent in nerves and was shown to produce distinctive PA microscopic signatures (Matthews et al., 2014). Nerves could also be visualized with hybrid PA/US systems in *ex vivo* preparations (Mari et al., 2015) and PAT in *in vivo* mouse models (Li R. et al., 2016). Imaging myelin in peripheral nerves might improve diagnostic accuracy if implemented in future clinical practice.

### *In vivo* Anatomy

Microscopic brain imaging *in vivo* is dominated by multiphoton detection of fluorescent sensors. Besides imaging neurons, multiphoton imaging has also been used for the quantification of vascular architecture. In particular, measuring the microvasculature diameter during sensory stimulation can estimate the cerebral blood volume (CBV) and clarify its relationship to neuronal activation (Kong et al., 2015; Kim et al., 2019). Label-free PA microscopes can image brain microvasculature by detecting absorption signals from individual red blood cells (Kong et al., 2015; Kim et al., 2019). An integrated microscope combining reflection-mode photoacoustic, multiphoton, and second harmonic detection was used to image the complex 3D neurovascular system of mice *in vivo* (Song et al., 2016). Although oxygen metabolism was not quantified in this study, the ability to image neuronal activity at a subcellular level and simultaneously record hemodynamic parameters would prove valuable in elucidating the underlying mechanisms of neurovascular coupling (Song et al., 2016).

Photoacoustic tomography *in vivo* is dominated by vascular contrast due to high hemoglobin concentrations that mask other photoabsorbers. Brain structures such as the third ventricle, superior sagittal sinus, and cerebral bridging veins were visualized *in vivo* (Razansky et al., 2011; Burton et al., 2013; Olefir et al., 2016; Chen et al., 2017). The carotid arteries and Willis Circle could be imaged with PA by illuminating through the oral cavity (Lin et al., 2015), delivering data similar to what, e.g., time-of-flight MRI techniques provide.

Anatomical imaging with a hybrid PA/US system enabled the correction of skull-induced artifacts in mice, facilitating microvasculature visualization with a field-of-view covering the entire cortex (Estrada et al., 2018). Imaging brain vasculature *in vivo* can also be achieved optically with short-wave infrared imaging (Bruns et al., 2017; Carr et al., 2018) and US (Errico et al., 2015). Both of these techniques feature several centimeters in penetration depth (Macé et al., 2011; Bruns et al., 2017) and excellent performance in capturing flow dynamics. In this configuration, photoacoustics could contribute valuable data on oxygen metabolism.

### Ischemia

Concerning pathological brain conditions, PA is naturally suited for studying brain ischemia due to its sensitivity to blood oxygenation. PAT systems lend themselves to label-free detection of stroke by recording an asymmetry in blood oxygenation. Furthermore, areas of compromised perfusion in which brain tissue may still be viable, the so-called penumbra, can be identified (Kneipp et al., 2014). Both OR-PAM and PAT were able to identify ischemia 3-5 min after onset. Hemorrhagic transformation could also be detected (Lv et al., 2020).

OR-PAM systems can be easily modified to enable localized photocoagulation by increasing the laser beams’ energy, such that small vessels can be occluded with high precision, leading to local ischemia (Hu et al., 2011a; Liao et al., 2013; Lv et al., 2020). After photocoagulation, ischemia caused a breakdown of the BBB, leading to extravasation of Evans Blue bound to albumin to visualize the extent of ischemia (Lv et al., 2020).

### Tumors

The ability to visualize anatomical structures based on contrast derived from exogenous and endogenous photoabsorbers makes PA imaging a valuable tool for non-invasive neoplastic disease monitoring, including brain tumors. Volumetric imaging of glioblastoma in nude mice was demonstrated without contrast agents by exploiting differential contrast under illumination with 800 and 850 nm. The authors also quantified tumor O_2_ saturation, which increased 15 days after inoculation, whereas the tissue became hypoxic 42 days after inoculation (Balasundaram et al., 2018). In another study, integrin expressing tumor cells were tagged with an IRDye800-based contrast agent while also assessing tumor SO_2_, which was 13% lower than in healthy brain tissue (Li M. et al., 2008).

Melanoma cells offer strong intrinsic contrast for PAI. Secondary melanoma brain tumors were monitored non-invasively with a reflection-mode PAM in small rodents (Staley et al., 2010). Circulating melanoma cells could be imaged in the brain of mice perfused with artificial cerebrospinal fluid, which was possible because melanoma cells naturally contain the highly absorbing pigment melanin. Still, high hemoglobin absorption prevented a transfer of the technique to *in vivo* detection (Deán-Ben et al., 2020). In addition to cell visualization, it is possible to successfully manipulate circulating B16F10 mouse melanoma cells with acoustic and photoacoustic pressure waves. Circulating cells in lymphatic vessels were trapped, focused, and redirected, showcasing how this method might be used for the non-invasive diagnosis of neoplastic disease (Galanzha et al., 2016). Identification of circulating melanoma cells in the superficial venous system of human subjects was also achieved with a PAT system. However, more extensive studies are needed to determine the value of including this imaging method in the staging of this neoplastic disease (Hai et al., 2020).

### Neurodegenerative Diseases

Amyloid plaque deposition is a hallmark of Alzheimer’s disease. Inflammatory processes and abnormal oxygen metabolism also play an important role in neurodegenerative processes and may be accessed by molecular imaging.

Positron emission tomography is considered the gold standard in Alzheimer’s plaque and inflammation imaging. Despite recent developments in simultaneous PET/MR acquisition, molecular information is limited to low spatial resolution in the 0.5–1 millimeter range (Zhang X. Y. et al., 2017). Optical imaging in preclinical settings has been facilitated by the development of NIR contrast agents targeting amyloid plaques (Tong et al., 2015). The plaques were visualized with an OR-PAM system after *i.v.* congo red injection, which is a classical photoabsorbing stain for amyloid. Plaque position, morphology, and relation to vasculature were imaged both *in vitro* and *in vivo* through a glass window. Again for this application, multispectral acquisition allowed blood vessels to be simultaneously imaged without contrast agent injection (Hu et al., 2009).

Inflammatory processes can be imaged with PA by using macrophage targeting probes such as CDnir7 (Kang et al., 2014). Alzheimer’s disease’s inflammatory component was quantified non-invasively *in vivo* using PAT detection after CDnir7 administration (Park et al., 2019).

Cerebral metabolic dysfunction has been shown to accompany plaque deposition in AD (de la Monte and Tong, 2014). PAT investigation of arcAβ mice showed a decrease in the cerebral metabolic rate of oxygen (CMRO_2_), while the oxygen extraction fraction (OEF) remained unchanged in control animals (Ni et al., 2018).

## Imaging of Brain Hemodynamics

A transient increase in neuronal activity leads to higher energetic demands in spatially confined regions of the brain. However, neuronal tissue does not possess significant energy stores (Magistretti and Pellerin, 1999; Shulman et al., 2001). Instead, nearby vessels dilate to ensure an adequate supply of glucose and oxygen, which are metabolized locally in neurons. This coupling of neuronal activity to cerebral blood flow (Huneau et al., 2015) is referred to as neurovascular coupling. Consequently, neuronal activation events can be inferred from observed hemodynamic signal changes via an estimate of a transfer function, the so-called hemodynamic response function (HRF) (Peng et al., 2019).

Functional imaging of the brain based on the neurovascular coupling is thus possible with essentially any imaging modality that can detect a transient change in blood volume, blood flow, or blood oxygenation. Here, we highlight the strengths and limitations of MRI, US, and optical imaging modalities to describe how PA can complement these modalities based on its sensitivity to blood flow dynamics and hemoglobin oxygenation.

Hemodynamic signal changes are famously imaged by fMRI, employing the so-called Blood Oxygenation Level Dependent (BOLD) contrast (Ogawa et al., 1990, 1992). BOLD is sensitive to changes in the concentration and oxygenation state of hemoglobin. The precise physiological mechanisms underpinning the BOLD contrast are, however, still not fully understood. Multimodal imaging systems which co-record fMRI data with PET (Aiello et al., 2016), EEG (Moeller et al., 2020), NIRS (Scarapicchia et al., 2017), and fluorescence imaging (Schwalm et al., 2017; Lake et al., 2020) are thus being employed to cross-validate the fMRI signals.

For preclinical experiments, fMRI is limited mainly to anesthetized measurements, although protocols for imaging awake mice (Chen et al., 2020), rats (Chang et al., 2016), and non-human primates (Chen et al., 2012) have been described. Temporal resolution for fMRI is limited to one imaging volume per second though trade-offs in spatial resolution enable line scanning at 10-20 Hz (Yu et al., 2014; Albers et al., 2018). Due to its non-invasiveness, fMRI has been extensively used to map functionally connected brain regions in humans (Maknojia et al., 2019).

Functional ultrasound interrogation and manipulation of brain networks have been proposed to overcome some of fMRI’s shortcomings in specificity, spatiotemporal resolution, and portability (Rabut et al., 2020). Cortical and deep thalamic sensory responses to stimulation were characterized by doppler-ultrasound imaging (Macé et al., 2011). Single-trial functional brain responses to stimulation in unconstrained behaving monkeys were recorded by ultrafast Doppler (Dizeux et al., 2019). Moreover, implantable ultrasound transducers combined with EEG allow more natural behavioral experiments to be carried out in mobile behaving rats (Sieu et al., 2015). A drawback for this modality derives from the fact that fUS imaging in human subjects is only possible in infancy before fontanelle calcification (Demene et al., 2017) or after skull removal for brain surgery (Imbault et al., 2017).

Optical imaging in the short wave infrared (SWIR), 1 to 2 μm in wavelength, facilitates hemodynamic measurements at depths greater than multiphoton microscopy. Highly detailed representations of the brain’s vasculature have been acquired after injection of indocyanine green (ICG) (Carr et al., 2018; Byrd et al., 2019), quantum dots (Bruns et al., 2017), and organic fluorophores (Wan et al., 2018), enabling the generation of detailed blood flow maps (Bruns et al., 2017). While this technique may enable deep brain functional imaging, further improvements in detector technology and molecular imaging sensors are necessary before widespread implementation.

Multispectral PAI can enhance the information gained from the techniques mentioned above through the non-invasive quantification of oxygenated (HbO) and deoxygenated (HbR) hemoglobin, total hemoglobin (HbT), and cerebral blood flow (CBF). From these parameters, the oxygen extraction fraction (OEF) and the cerebral metabolic rate of oxygen (CMRO_2_) can be calculated. Photoacoustic microscopy has been used to investigate the hemodynamic coupling at the microvasculature scale by OEF and CMRO_2_ estimation (Liao et al., 2010, 2012; Yao et al., 2015; Cao et al., 2017). OR-PAM has also made important contributions to our understanding of the physiological underpinnings of the BOLD signal in fMRI. An increase in HbR in cerebral arterioles was observed hundreds of milliseconds after electrical paw stimulation, which likely corresponds to the initial decrease in BOLD signal commonly referred to as the ‘initial dip’ (Liao et al., 2012).

Although a hybrid multiphoton and photoacoustic microscope has been presented (Song et al., 2016), this was mainly used for structural imaging. Simultaneous recordings of genetically encoded fluorescent calcium indicators and PAI of hemodynamic parameters could shed light on individual contributions of neurons and glial cells to CBF increases (Muñoz et al., 2015). To achieve these results, OR-PAM techniques usually still necessitate scalp and sometimes also skull removal and are limited to a penetration depth of about a millimeter (Hsu et al., 2018).

Because of the limitations of OR-PAM, tomographic PAI techniques have also been employed for imaging neuronal activation via neurovascular coupling. Photoacoustic signal enhancements localized to large cortical blood vessels in response to sensory stimulation have been reported (Wang et al., 2003a). In addition to cortical responses to sensory stimulation (Zhang et al., 2018; Olefir et al., 2019), thalamic signals deeper in the brain also seemed to be detectable by PAT (Zhang et al., 2018). Brain hemodynamic imaging with a PAT system revealed functional brain networks in a centrally situated coronal brain slice in mice (Olefir et al., 2019). The whole mouse brain can theoretically be captured by this imaging technique, though covering the entire brain volume requires moving either the transducer array (Wang et al., 2003b; Zhang et al., 2018) or translating the subject (Burton et al., 2013; Olefir et al., 2016).

Volumetric PA acquisition for whole-brain imaging is desirable and can be achieved by using an ultrasound detector array mounted on a hemispherical surface (Gottschalk et al., 2019b). Light is delivered via an optical fiber bundle situated in a central opening in the transducer array. Such a configuration allows for 100 Hz volumetric imaging, with variable wavelengths on a per laser pulse basis, with a spatial resolution of 175 μm (Chen et al., 2017). Practically, spectral unmixing of HbO, HbR, and HbT require data acquisition after illumination at 506, 540, 560, 575, and 585 nm wavelengths, which limits the effective temporal resolution after unmixing to 20 brain volumes per second. After electrical paw stimulation, 31.4, -3.5, and 6.6 percentage changes in HbO, HbR, and HbT relative to baseline were observed (Mc Larney et al., 2020).

Hemodynamic PAI in awake, behaving rats has also been reported. 3D data acquisition was performed with an implantable miniaturized three-layer transducer array providing a field-of-view that covered the entire brain. After presenting optical stimuli, CBV was quantified in the visual cortices and the superior sagittal sinus showing a perfusion increase after stimulation (Tang et al., 2016). However, challenges in miniaturization remain for this approach and implantable microscopes or mobile ultrasound detectors represent cheaper and more practical options.

### Epilepsy

Hemodynamic imaging also lends itself to the study of pathologies that induce massive neuronal activation followed by a hemodynamic response such as epileptic seizures. Proof of principle work in this pathology has been performed with spherical arrays in rats (Wang et al., 2012) while seizures in awake animals were imaged using portable sensor arrays (Tang et al., 2015). PAT helped to characterize the spatial spread of pharmacologically induced seizure activity in the mouse (Zhang et al., 2018) and could also discern patterns related to seizure activity in thalamic regions beyond the reach of optical imaging (Gottschalk et al., 2017). Tissue perfusion changes showing increased blood flow in seizure foci measured with PA are also in agreement with optical imaging spectroscopy studies (Harris et al., 2014).

As accessible as the neurovascular coupling is for many imaging methods, it only provides a rough estimation of the metabolic activity in a given brain region. Thus, it is probably biased toward energy-intense presynaptic neurotransmitter recycling (Logothetis, 2008; Gauthier and Fan, 2019), while it can not differentiate between excitatory or inhibitory neurotransmission. This contrast mechanism can also not resolve individual cells or the timing of action potentials, which can be accessible with molecular contrast agents.

## Molecular Contrast for Photoacoustic Neuroimaging

Molecular bioimaging aims to non-invasively map biological structures and monitor cellular processes at the molecular level. Molecular contrast agents convert a specific molecular structure or process of interest into an appropriate signal for a given imaging method, which can otherwise not obtain this information. If the signals from the molecular contrast agents can be made sparse, the molecular information can also be localized at a resolution that exceeds that dictated by the imaging device’s point-spread-function (Betzig et al., 2006). In this regard, molecular contrast agents amplify and magnify molecular information.

This section addresses the most promising contrast mechanisms currently available for PAI in neuroscience.

### Desired Properties of Molecular Contrast Agents for PA

For PA imaging, the building blocks for contrast agents ideally fulfill the following set of photophysical properties: a large molar extinction coefficient at wavelengths at which tissue absorbance is relatively low such as in the near-infrared window, a distinct absorption peak to facilitate spectral unmixing, a low quantum yield for maximum light conversion to thermal energy via non-radiative decay, and a high photostability (Weber et al., 2016).

Concerning the interaction with the biological tissues of interest, a molecular contrast agent should also possess high stability in biological media such as blood and cerebrospinal fluid, low toxicity, and immunogenicity. For neuroimaging, the contrast agent’s size and charge should also enable permeability through the blood-brain-barrier (BBB) (Milej et al., 2017), such that techniques to open the BBB do not have to be employed.

**FIGURE 2:**
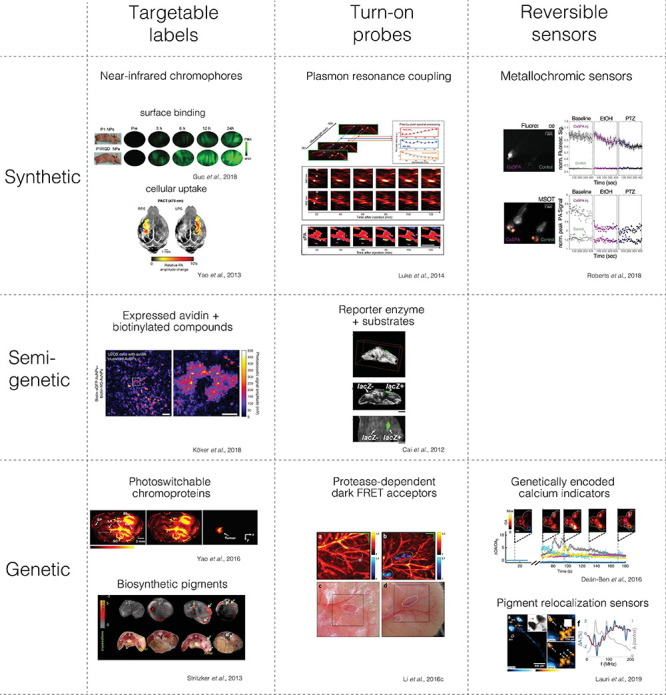
Main classes of molecular contrast agents with examples from photoacoustic imaging. The figures were adapted from the references with permission from the authors.

There is a wide variety of contrast mechanisms applicable in preclinical neuroimaging. It is thus useful to differentiate between the following molecular contrast agent classes with respect to which molecular state or process they indicate: (i) targetable labels that can map the biodistribution of a target structure, (ii) turn-on probes, whose signal can be irreversibly activated by a molecular interaction, and (iii) reversible sensors, which can dynamically adopt different signaling states in response to an analyte of interest (Figure 2, columns).

Based on their composition, we can also differentiate synthetic from semi-genetic or fully genetic contrast agents (Figure 2, rows).

Synthetic agents are produced through chemical and nanotechnological methods. Semi-genetic contrast agents have a genetically encoded component that interacts with at least one synthetic component. In distinction, genetic contrast agents are generated via genetic programming of a biosynthetic pathway within a cellular (or cell-free) biomolecular machinery, which may require endogenous co-factors.

The molecular contrast agent type has, for instance, implications for the delivery method that can be used. Options for delivering contrast agents to the brain include intravenous, intrathecal, intracerebroventricular, or intracerebral injections.

For intravenous delivery, a prolonged circulation time may be desired for imaging blood flow and contrast agent accumulation in the target tissue.

To overcome the intact BBB, high lipid solubility and a particle size under 1 nm are advantageous (Abbott and Romero, 1996; Pardridge, 2005). In neoplastic disease, BBB disruption allows the passage of larger particles up to 100 nm (Houston et al., 2020). Transport processes across the BBB can be exploited by incorporating suitable modifications that enable receptor-mediated transcytosis (Pulgar, 2018).

The BBB can also be temporarily opened using focused ultrasound, thus increasing the spectrum of compounds that can be efficiently delivered to the brain (Chen et al., 2019).

An intracranial injection is an alternative option for the direct delivery of dyes, therapeutics, or viruses to the brain. This method’s limitations are that only a small volume can be administered (usually around 1–2 μL) at low injection rates, usually not exceeding 0.1 μL/min to avoid damaging the brain (Cetin et al., 2006). Alternatively, surgically implanted osmotic pumps can dispense defined volumes over longer periods (Sepúlveda et al., 2012).

Whereas synthetic agents have to be delivered to the tissue of interest and then targeted to extra- or intracellular structures, genetically encoded contrast agents have the advantage that they can be produced directly in genetically defined cells conditioned on specific cellular states. Transferring the necessary genetic information can be achieved by germline transgenesis or via the administration of viral (Sung and Kim, 2019) or non-viral (O’Brien and Lummis, 2006) DNA or RNA delivery vehicles.

Gene expression via viral transduction is preferred *in vivo* and can be achieved through interstitial injection of neurotropic viruses or systemic vector administration (Foust et al., 2009; Manfredsson et al., 2009; Challis et al., 2019). Popular vectors include Lentiviruses, Herpes Simplex, or Adeno-associated viruses (Cetin et al., 2006; Challis et al., 2019; Ingusci et al., 2019). The selective use of gene promoters allows for an increasing number of neuronal or glial cell types to be specifically targeted. After infection, the contrast agent DNA sequence can be integrated into the host genome (Vargas et al., 2016), or it persists as an episome in the nucleus (Haggerty et al., 2020). Regardless of DNA integration, because neurons do not divide, long-term contrast agent expression can be achieved.

Besides, the knowledge that different neurotropic viruses have a preferred direction of transport within infected neurons can be exploited to identify or target the pre- or post-synaptic connections of a neuronal population of interest (Saleeba et al., 2019).

### Intravascular Contrast Agents

The specific biodistribution of contrast agents in predefined compartments of the body can reveal local anatomical and functional aspects. Examples include “blood pool agents” that are constrained to the vascular system, nanomaterials that accumulate in tumors or post-stroke tissue due to enhanced permeability and retention effects (PRE) (Nel et al., 2017), or leakiness of the BBB.

At a finer scale, information can be gained from targetable labels that can be directed to specific cells via surface affinities or selective cellular uptake (see next section).

Many synthetic photoabsorbers can serve as blood-pool agents and as the building blocks for targetable agents in PAI: organic contrast agents (e.g., organic dyes), conjugated polymer nanoparticles, and other inorganic nanostructures (e.g., carbon nanoshells).

Small molecular dyes can visualize blood vessels and enable perfusion measurements in healthy brains and brain tumors (Burton et al., 2013). Strong photoabsorbers such as black paramagnetic polyethylene microspheres (Luís Dean-Ben and Razansky, 2018) or oil-dissolved IR-780 dye droplets (Zhang P. et al., 2019) were used for localization microscopy with photoacoustics to go beyond the diffraction limit. Applying these techniques to PA angiography enables high-resolution maps of brain vasculature to be acquired *in vivo* (Zhang P. et al., 2019).

Advances in material science have led to the creation of functionalized synthetic nanoprobes, which can be tuned for high absorption in a defined spectral range, exhibit resistance to photobleaching, and be functionalized with the addition of dyes or drugs. Most synthetic nanoparticles developed for photoacoustic applications have been employed for studying brain tumors: noble metal nanoparticles (Shang et al., 2017), copper nanoparticles (Wu et al., 2018; Zhang H. et al., 2019), carbon nanorods (Qian et al., 2018), iron-based nanoparticles (Zhou et al., 2018), MoS2 nanosheets (Chen et al., 2016). Nanoparticles that enable multimodal imaging have been a focus of recent research: PA/MRI/Raman (Kircher et al., 2012), PA/MRI/PET (Fan et al., 2014), PA/MRI (Zhu et al., 2015; Qiao et al., 2018). Circulating dyes and nanoparticles have been shown to accumulate in brain tumors due to a disruption of the BBB and PRE (Kircher et al., 2012; Burton et al., 2013; Fan et al., 2015; Neuschmelting et al., 2018). However, these contrast agents are rapidly cleared from the bloodstream by the reticuloendothelial system and the liver.

### Targetable Labels

#### Synthetic Targetable Labels

Strategies to achieve specific targeting of cellular structures as opposed to just relying on PRE include contrast agents binding to endothelial tumor cells (Ray et al., 2011), transferrin receptors (Zhu et al., 2017), adenosine receptors (Liu et al., 2019), and integrin (Guo et al., 2018). Additionally, the photoabsorbing properties of PA contrast agents make them suited for hyperthermia treatment in glioblastoma (Manivasagan et al., 2017; Zhu et al., 2017; Balasundaram et al., 2018; Guo et al., 2018; Liu et al., 2019) (Figure 2, upper left segment).

A good example for a targeted cellular uptake is 2-NBDG, a fluorescent 2-deoxyglucose variant, which in analogy to the well-established method for PET, can be internalized preferentially into cells that have an increased energy demand. Once taken up by the glucose transporter, the glucose analog is phosphorylated by cellular kinases such that it cannot leave the cell. Using this compound in PA imaging, it was possible to visualize increases in brain glucose metabolism after sensory stimulation and quantify hemodynamic brain responses in the same experiment using two excitation wavelengths (Yao et al., 2013).

Contrast agents may also be targeted to selective cells *ex vivo*, which are then injected into an organ(ism). In this mode, the cell itself can be considered the contrast agent, and its interactions in the tissue can be monitored. Mesenchymal stem cells labeled with Prussian blue were injected intravenously and used to monitor the extent of the healing process after traumatic brain injury (Li et al., 2018). Similarly, tumors can be targeted for imaging using nanoparticle-labeled mesenchymal stem cells (Qiao et al., 2018). However, synthetic labels have the disadvantage that they are rapidly diluted upon cell divisions, and that contrast will also persist even if the cell has already died, shortcomings that can be alleviated with genetic labels.

#### Semi- and Fully Genetic Targetable Labels

Whereas fully-synthetic targetable labels depend on a selective targeting mechanism that has to exploit a particular cell surface property or a selective uptake mechanism, genetic targeting mechanisms can provide selectivity by tapping into the specific genetic program of particular cells (Figure 2, middle and bottom row).

The genetic component can be stably expressed in germline transgenic animal models such that the contrast can be imaged without further intervention. Alternatively, the genetic information for encoding the label can also be introduced to an organism via (non-)viral carriers or via delivering genetically modified cells. In all cases, the contrast can be made dependent on the genetic program of selective cells, e.g., by using specific promoters. The genetic label also propagates to daughter cells without loss and is also selective for viable cells as their expression relies on an intact cellular metabolism.

Semi-genetic targeting of a synthetic label can, for instance, be achieved by expressing an affinity handle, e.g., on the cell surface. In one such system, biotinylated gold-nanoparticles were bound to avidin expressing cells in culture (Köker et al., 2018) or lung cancer cells in a rodent model (Lee et al., 2018) and detected by PA.

As pertains to fully genetic labels, fluorescent proteins such as mCherry have been shown to give rise to photoacoustic contrast (Razansky et al., 2009). However, the absorbance peak in the visible spectrum competes with strong photoabsorbance from hemoglobin *in vivo* and thus limited PA imaging to zebrafish and fly pupae. Moreover, chromoproteins consisting of chromophores built only from aminoacids via cyclization reaction have generally low photostability, as is the case for GFP derivatives.

In comparison, chromoproteins, which bind small biosynthetic chromophores such as biliverdin, exhibit higher photostability and absorbance peaks that reach further into the near-infrared window than red proteins such as E2-Crimson, mNeptune, mKate2, eqFP670, and TagRFP65, resulting in superior photoacoustic signals (Filonov et al., 2012). For instance, IRFP expressing U-87 MG cells could be detected after cerebral injection into nude mice (Deliolanis et al., 2014).

Another powerful strategy to increase the contrast of fully-genetic labels is to use reversibly switchable fluorescent proteins (RSFP), which can be switched into a non-fluorescent dark state by laser illumination accompanied by a shift of the absorption spectrum (photochromism) (Figure 2, lower left segment). This photophysical property allows for photocontrol of distinct time-varying signals (Marriott et al., 2008), which can be temporally unmixed reliably from each other and from strong static background absorbers such as hemoglobin in PAT even if they are spectrally overlapping (Deán-Ben et al., 2015b; Stiel et al., 2015). By expressing switchable bacteriophytochrome (BphPs) proteins with absorbance spectra in the near-infrared, tumor cells could be differentiated from background in mouse brain ∼3 mm beneath the scalp surface subdiffraction imaging of individual tumor cells (Yao et al., 2016). Expression of BphPs also allowed for the segmentation of tumor cells from subcutaneous tumor vasculature *in vivo* (Märk et al., 2018) and differentiating bacteria injected in tissue (Chee et al., 2018). Expression of selected truncated BphP variants optimized for extinction coefficients, switching kinetics, and reduced photofatigue allowed for the segmentation of as few as 500 cells/ul Jurkat T lymphocytes after subcutaneous injection into mice via classification of a set of features extracted from the photoswitching signal trajectories (Mishra et al., 2020).

In distinction to chromoproteins, biosynthetic pigments, i.e., small molecular dyes synthesized by enzymatic processes, can have a high photostability. Each translated enzyme performs many rounds of catalysis, producing numerous photoabsorbers, thus leading to a substantial amplification factor of the genetically defined process.

Melanin is a polymer derived from the amino acid tyrosine as a substrate for the enzyme tyrosinase. Melanin offers excellent contrast in PA imaging, as is well-known from skin imaging of melanin-containing nevi and ephelides. Early on, tyrosinase has thus been used as a reporter enzyme for photoacoustics (Krumholz et al., 2011; Stritzker et al., 2013). However, Melanin precursors have cytotoxic properties if they are not sequestered in melanosomes, membranous compartments found in specialized cells (Raposo and Marks, 2007). A Tet-On inducible tyrosinase reporter-gene system was expressed in tumor cells, which were implanted *in vivo*. Upon doxycycline administration, melanin production could be initiated, and this process could be visualized *in vivo* with PA imaging (Paproski et al., 2014). The inducible gene expression can control the duration and toxicity to some degree, but more delicate cells such as neurons may still be substantially compromised. Another solution was found by expressing self-assembling nanocompartments that can accept guest molecules to their lumen and thus sequester enzymatic processes. Specifically, tyrosinase was targeted to the inner surface of so-called encapsulins from the bacterium *M. xanthus*, which assemble to semi-permeable icosahedra that would allow the substrate tyrosine but sequester the toxic melanin polymer. Consequently, melanin production was confined to the lumen of the engineered nanomelanosomes, producing strong PAT signal but shielding the cells from toxicity-induced damage produced by non-encapsulated tyrosinase (Sigmund et al., 2018). It will be exciting to see combinations of these labels with recently developed gene reporters for US (Farhadi et al., 2019).

### Turn-On Probes

An example of fully synthetic turn-on PA contrast agents are BBB-permeable aniline compounds that strongly increase absorbance and thus PA signal around 750 nm upon chelation with Cu^2+^ and may thus be useful for imaging copper accumulation in brains that may be correlated with amyloid plaque formation (Wang et al., 2019). Furthermore, gold nanoparticles conjugated to antibodies have been shown to change their PA spectra upon target binding due to plasmon resonance coupling (Luke et al., 2014).

One of the first semi-genetic turn-on mechanisms adapted for PA imaging is the well-known reporter enzyme β-galactosidase, which converts the soluble synthetic substrate X-gal into a blue precipitate. Delivery of the X-gal substrate to β-galactosidase-expressing cells in culture was shown to generate PA contrast from the resulting precipitate upon 650 nm illumination (Li et al., 2007). Simultaneous detection of the microvasculature and lacZ labeled tumor cells via AR-PAM imaging was useful in identifying tumor feeding vessels (Li L. et al., 2008). Individual LacZ labeled cells could be imaged with an OR-PAM setup showing intracellular contrast agent distribution, and PAT methods were able to detect LacZ labeled cells up to a depth of 5 cm in biological tissue (Cai et al., 2012).

Förster resonance energy transfer (FRET) entails energy transfer from a donor fluorophore to an acceptor chromophore, which can also alter the corresponding PA signals (Wang and Wang, 2012; Wang et al., 2013) (Figure 2, bottom of middle column). An activatable PA reporter for protease activity was explored *in vitro* using genetically expressed chromoproteins with low QY as a dark FRET acceptor. The authors expressed ultramarine variants linked to EGFP as a FRET donor via a caspase-3 cleavage site (Li Y. et al., 2016). When caspase-3 activity was induced in mammalian cell culture, an increase in EGFP fluorescence was observed due to the loss of FRET upon proteolytic separation of the FRET pair. When FRET-pairs were purified and measured by PA without and with proteolytic cleavage of the linker, a decrease in the PA signal was observed consistent with an increase of donor fluorescence. Modulation of FRET from a dark acceptor could be an interesting PA reporter mechanism, especially if genetically controlled FRET donors with high QY in the near-infrared could be developed.

### Dynamic Sensors

As discussed above, volumetric PA imaging is a scanless technique that can pick up signals with higher temporal resolution and from deeper tissue layers than multiphoton techniques. Thus, it is an attractive method for mapping dynamic and distributed brain processes. As laid out above, PAI can map neurovascular coupling dynamics at adequate temporal sampling rates and over large portions of the brain.

However, it is desirable to obtain more specific information about the cellular and molecular identity and temporal profile of neuronal activity.

Patch-clamp measurements of neurons naturally provide the best spatial and temporal resolution for characterizing neuronal electrical activity (Petersen, 2017), but it is often valuable to capture the propagation of charge fluxes over many cells of specific identities.

Imaging electrical events in neurons was first achieved with electrochromic sensors, in which membrane potentials cause changes in absorption (Tasaki et al., 1968; Ross et al., 1977; Grinvald et al., 1981; Grinvald and Hildesheim, 2004). Despite the low signal changes of usually less than 1%, impressive imaging results allowed millisecond activation maps resolving individual columns in the visual cortex, a highly influential finding for the field of visual perception (Sharon and Grinvald, 2002).

While synthetic voltage dyes also tend to stain glia, genetically encoded voltage-sensitive indicators (GEVIs) can be targeted to selective neuronal types. Several types of GEVIs have been realized based on fusions to voltage-sensing domains or rhodopsins, which can be read out by FRET or QY changes. They have become brighter and increasingly red-shifted, enabling the study of subcellular features of the action potential waveform (Xu et al., 2017; Bando et al., 2019; Panzera and Hoppa, 2019). Challenges persist in applying this technology *in vivo* as heavily convoluted neuronal cells make it difficult to accurately record subcellular signals with sufficient speed and spatial resolution to avoid partial volume effects. Despite these technical challenges, all-optical electrophysiological (optophysiological) studies have begun to provide insight into the circuit activity underpinning behavior (Adam et al., 2019).

There have been initial attempts to pick up voltage changes by PA via fluorescence quenching of voltage-dependent dyes, which should increase PA intensity (Zhang H. K. et al., 2017). The fluorescent voltage sensor dipicrylamine was imaged with OR-PAM in HEK cells *in vitro* and PAT *in vivo* after administration of chemoconvulsants (Rao et al., 2017). Similarly, the voltage sensor VSD IR780 perchlorate was imaged *in vivo* after seizure induction (Kang et al., 2019). While VSD fluorescence changes were used as a positive control in these studies, *in vivo* ground-truth electrophysiology during more physiological stimulation paradigms would be desirable to assess these contrast agents’ potential.

Given the small signal change and the limited concentration and slow replacement of photobleached voltage sensors in the cell membrane, PA detection of voltage changes will remain challenging but very desirable to advance fast volumetric voltage imaging.

Calcium transients are too slow to identify the precise timing of action potentials, such as synchronicity between AP trains (Kwan and Dan, 2012; Hoang et al., 2020). However, calcium dynamics lie within the temporal resolution of many current imaging methods, and estimating action potentials by deconvolution is possible under certain conditions (Pachitariu et al., 2018; Hoang et al., 2020). Furthermore, intracellular calcium concentrations can change by two orders of magnitude in response to neuronal spiking (Schiller et al., 1995; Ali and Kwan, 2019) and can be measured in the cytosol and nucleus of the cell, where higher concentrations of calcium sensors can be achieved than possible for membrane-bound voltage sensors.

Volumetric PAI at current frame rates up to 100 fps is better suited for recording Ca^2+^ transients than action potentials due to the slower kinetics of calcium sensors (Chen et al., 2013).

Canonical calcium sensors include synthetic dyes such as Oregon Green 488 Bapta-1 AM-ester, which can reach the cytosol, where endogenous esterases cleave the AM-ester to activate and trap the sensor inside cells (Tsien, 1980, 1981; Stosiek et al., 2003). As for genetically encoded calcium indicators (GECIs), a design based on GFP fused to calcium-binding protein CaM, and its binding peptide (GCaMP) has been substantially optimized over two decades to yield robust tools for routine measurements (Miyawaki et al., 1997; Chen et al., 2013; Yang et al., 2018). Furthermore, ratiometric calcium sensors have been built by crosslinking FRET pairs via a calcium-triggered contractile element from muscle fibers (Mank et al., 2008; Trigo-Mourino et al., 2019).

As a step to enable intracellular calcium imaging with photoacoustics, a synthetic calcium sensor for photoacoustics (CaSPA) was recently designed to selectively respond with a robust blue-shift of the spectrum (metallochromism) upon calcium binding while minimizing quantum yield and ensuring high photobleaching resistance (Roberts et al., 2018) (Figure 2, upper right segment). Due to its esterified BAPTA moiety (Tsien, 1981), the compound could be delivered into cells, organoids, and into zebrafish and exhibited high selectivity. CaSPA is based on a semi-cyanine chromophore that can be modified to red-shift the absorbance spectrum, which is an obvious next goal to separate it from the brain activity-dependent hemoglobin absorbance in the visible range.

It could also be recently shown that GCaMP can be detected by PAI, which was chosen because it exhibits a strong change in absorbance instead of calcium sensors that work by a change in quantum yield (Deán-Ben et al., 2016) (Figure 2, lower right segment). In proof-of-principle experiments, simultaneous fluorescence, and photoacoustic recordings were conducted in GCaMP-expressing zebrafish larvae and *ex vivo* adult zebrafish brains using chemical calcium triggers. These results have been corroborated by an *ex vivo* murine brain preparation showing signal changes from GCaMP6f in response to an activating chemoconvulsant (Gottschalk et al., 2019a).

As with synthetic calcium sensors, one obvious path for improvement will be to develop red-shifted calcium-dependent chromoproteins, e.g., BphPs, as detailed above. For example, NIR-geco1 exhibits absorbance and emission maxima at 678 and 704 nm, respectively (Qian et al., 2019). However, the protein is only moderately metallochromic, and *in vivo* paw stimulation experiments resulted in only a 0.3% fluorescence signal decrease in the somatosensory cortex of mice (Qian et al., 2019), while no photoacoustic signal changes were shown. Another recently developed genetically encoded Ca^2+^ sensor, iGECI, is a NIR Förster resonance energy transfer (FRET)-based calcium indicator with a fixed maximum absorbance at 670 nm. *In vivo* imaging measurements yielded a negative 3% change in fluorescence after stimulation. The authors report simultaneous iGECI fluorescence and PA imaging of hemodynamics but no direct PA detection because PA signals are likely too small to exceed the noise (Shemetov et al., 2020). Further, NIR Ca^2+^ sensors like GAF-CaMP2 have also been reported to have modest absorbance change and have only been validated in cell culture (Subach et al., 2019).

None of the constructs listed above has the necessary properties for successful application in PA imaging of calcium transients in the NIR. One way to mitigate this problem might be to develop a reversibly switchable calcium sensor, applying some of the strategies illustrated in the section on reversibly switchable photochromic proteins above, and use more complex signal acquisition and data processing schemes to derive calcium dynamics. Research in this area is intense as, apart from the application in PA imaging, NIR Ca^2+^ sensors would be valuable tools for multiplexing with other visible range Ca^2+^ reporters and optogenetic actuators, enabling more complex neurophysiological experiments. Ca^2+^ imaging with PA could also benefit from progress made in image acquisition hardware and new computational strategies for spectral unmixing and image reconstruction.

A helpful step toward developing such a sensor would include the standardization of PA imaging setups and protocols for high-throughput screening of biosensor candidates (Hofmann et al., 2019).

A central signal transduction pathway in neurons, for which molecular sensors are desired, are GPCRs. GPCRs are a large family of cell surface receptors that interact with a wide variety of external signals, which regulate a myriad of cellular functions (Gurevich and Gurevich, 2019). This function makes them one of the most attractive targets for pharmaceutical intervention. Binding of an extracellular ligand to a GPCR results in G-protein activation, followed by signaling through the cAMP or phosphatidylinositol pathway, which releases intracellular calcium.

This signal transduction chain was used to generate GPCR-reporter cells that express a specific GPCR together with a calcium indicator, such that a calcium-induced fluorescence signal change answered the presence of a specific ligand. By implanting these whole-cell sensors into the brain of mice, dopamine and norepinephrine release could be monitored via fluorescence (Muller et al., 2014).

Direct fluorescence sensors for GPCR activity were designed in analogy to GCaMP by fusing circularly permuted fluorescent proteins to GPCR, which translate conformational changes in the GPCR to the fluorescent protein, altering the fluorescent signals (Patriarchi et al., 2018; Sun et al., 2018). Although these sensor mechanisms may, in principle, also be accessible by photoacoustics, the current wavelength range is far from optimal, as discussed above.

Recently, a different PA contrast mechanism was introduced based on the reversible relocalization of biosynthetic pigments (Lauri et al., 2019). To turn the static pigments into dynamic contrast changes, the authors took inspiration from cuttlefish, which can change their skin’s photoabsorbing patterns by rapidly contracting or dispersing pigment-filled compartments within specialized chromatophore cells via GPCR-induced calcium influxes. As a biomimetic analogon, cells containing melanin-filled melanosomes from *Xenopus laevis* were transplanted into the midbrain of juvenile zebrafish. The strong PA contrast enabled RSOM (an AR-PAM implementation) to resolve the contraction of individual melanophore cells in response to ligand-induced GPCR activity. The pigments’ contraction and dispersion could also be read out by a change in photoacoustic signal frequency as an additional observable parameter for molecular photoacoustics. This molecular sensor mechanism could be generalized by genetically reprogramming other cell types to relocalize pigments via various mechanisms (Figure 2, lower right segment).

## Toward Translation to Clinical PA Neuroimaging

PA imaging has already found applications in thyroid imaging, breast imaging, dermatology, and sentinel lymph node detection (Steinberg et al., 2019). The main obstacle for neuroimaging with PA in humans is the skull, which constitutes a barrier to both excitation light and the emitted ultrasound.

*Ex vivo* PA imaging through the intact skin and skull was attempted in primates and visualized the superior sagittal sinus and prominent bridging veins (Yang and Wang, 2008). The same group also attempted imaging a phantom through a human skull and could at least detect major gyri from an excised canine brain (Nie et al., 2012).

An exciting opportunity, which has already been seized by US, is to image through the fontanelle in neonates’ skull, which offers an acoustic window as the skull is not yet calcified. In clinical diagnosis, transfontanelle US imaging can be used to rule out hemorrhages, as well as hypoxic brain injuries (Dudink et al., 2020). Apart from these implementations in anatomical imaging, functional neonatal ultrasound in newborns proved useful for identifying ictal foci by tracking hemodynamic responses to massive brain activation during seizures (Demene et al., 2017). Notwithstanding, MRI is still the preferred modality in the absence of an absolute contraindication, US being used mainly as a screening tool at the bedside and for disease follow-up.

Initial phantom experiments have been conducted to ascertain the feasibility of transfontanelle PA imaging (Wang et al., 2008). In a sheep model with a cranial window, combined imaging with US and PA could detect experimentally induced intraventricular and intraparenchymal hemorrhage (Hariri et al., 2017). It remains to be determined whether PA detection may substantially outperform the cheaper and more available stand-alone US systems in diagnostic accuracy for these pathologies.

PA may also play a role in the operating theater to identify structures that should not be lesioned or help determine the tumor-free resection margins. However, a technical challenge that needs to be overcome is to realize acoustic coupling of the brain to the US detector in a non-invasive and convenient way. In this context, recent contact-free realizations of PAM seek to detect the photoacoustic effect via a change in the refractory index, read out via a co-focused laser beam (Hajireza et al., 2017). This method for non-contact reflection mode PA sensing showed label-free image contrast similar to the established hematoxylin and eosin staining in a proof-of-principle study. There is ongoing research to apply this detection method also for imaging fresh brain samples and *in situ* implementations during surgery (Abbasi et al., 2019; Ecclestone et al., 2020). Significant challenges to implementing such an imaging strategy *in vivo* in the operation theater are represented by insufficient imaging depth at the low wavelengths (∼250 nm).

Another possible implementation of intraoperative PA neuroimaging refers to the discrimination of peripheral nerves from the surrounding tissue. Recent phantom studies indicate superior contrast of peripheral nerves can be achieved with interventional PA/ultrasound devices as opposed to stand-alone US imaging system (Matthews et al., 2014; Mari et al., 2015), as also supported by *in vivo* experiments in mouse models (Li R. et al., 2016). Furthermore, diabetic polyneuropathy was found to cause significant increases in cross-sectional area in the median and sural nerves and decreased blood volume in PAI, which may indicate a possible advantage of combined PA/US over US alone (Yagihashi, 2018).

## Conclusions

Rapid progress in laser technology and ultrasound detector arrays have allowed for a set of PAI systems, which address brain imaging at different temporal and spatial scales and effectively complement more established imaging techniques.

PA combines the advantages of mapping the biodistribution of photoabsorbers via multispectral illumination with the lower scattering of ultrasound to obtain informative resolution in tissues at depths beyond the reach of multiphoton detection.

In the rodent system, still probably the most frequently used model system in neuroscience, volumetric PA acquisition can also capture the whole brain but faster than MRI. However, *in vivo* differentiation of anatomical brain structures is currently not as detailed as in preclinical MRI, where cortical column mapping at ∼20 micrometer isotropic resolution can be achieved (Wang et al., 2020).

Given that the rodent brain’s geometry is quite similar between animals and much more static than, e.g., internal gastrointestinal tissue, anatomical resolution, and spectral unmixing can be rapidly and substantially improved by further refinement of reconstruction routines. This process can be supported by information on skull anatomy (Estrada et al., 2018), correction for photon flux, and correlation to complementary modalities (Attia et al., 2016; Gehrung et al., 2020), aided by machine-learning (Reiter and Lediju Bell, 2017; Davoudi et al., 2019).

Furthermore, PA sensitivity to hemoglobin absorption enables label-free quantification of brain oxygen metabolism in addition to fast detection of blood flow also accessible to ultrasound and SWIR imaging using blood-pool agents.

With ML-assisted unmixing algorithms, volumetric PA can deliver video-rate maps of oxygenation and blood volume from rodent brains (Olefir et al., 2020). Similar information can be inferred from MRI via modeling based on prescans and oxygenation challenges. These MRI data will be available at lower volume rates but already from humans. The native sensitivity of PA for hemodynamics is also proving to be beneficial to further investigate the physiology of neurovascular coupling via combined *in vivo* PA/multiphoton microscopes, which can improve the interpretation of macroscopic hemodynamic data. It is also conceivable to measure location-specific hemodynamic response functions (Lee et al., 2010) in multispectral PA by sequential electrical or optogenetic stimulation to improve hemodynamic neuroimaging fidelity by PA.

Concerning *ex vivo* PA imaging of the nervous system, a few tissue components such as neuromelanin, lipids, cytochrome, nucleic acids could be localized to provide more histological context before more complex fixation and staining protocols are carried out.

Notwithstanding these promising results, most whole-brain functional PA data has been acquired with custom-built setups, and the imaging equipment has yet to be made available to the broader neuroscience community.

A substantial increase in the value of PA imaging for preclinical neuroscience is also expected from the next generation of dynamic molecular sensors that will provide robust PA signals in response to brain activation events. There still seems to be a wide-open field for synthetic chemists to optimize NIR compounds for PA detection, which is an attractive application of these compounds given that the quantum yield in this wavelength range is already relatively low. However, in addition to optimal photophysical properties, equal attention must be paid to optimizing biodistribution. Solutions must be developed to target and deliver the compounds to the desired brain cells and into the relevant cellular compartments, such as the cytosol, while preserving their functionality. This subcellular targeting can be particularly challenging for synthetic nanostructures such as gold nanoparticles or nanotubes, although they may have superior extinction coefficients in the NIR range. Thus, innovative strategies for cellular targeting and delivery are needed that can successfully include semi-genetic approaches relying on mechanisms like enzymatic cleavage or intracellular aggregation.

Concerning genetically encoded contrast agents, further improvements to far-red shifted chromoproteins, that will ideally also be photoswitchable, will deliver more suitable candidates for molecular neuroimaging. Besides, there are ample opportunities to genetically control biosynthetic pigments as knowledge on multi-enzymatic biosynthetic pathways becomes available.

For behavioral neuroscientists, a manifest goal for mesoscopic, volumetric imaging techniques is to directly and non-invasively record and modulate neuronal activity *in vivo*. As discussed above, recent progress has been made in the development of reporters for molecular ultrasound (Maresca et al., 2018; Farhadi et al., 2019) and MRI. While these two modalities rely on well-characterized, mature technology that has already found its way into the neuroscience toolkit, the combination of fast volumetric PA imaging and robust sensors for neuronal activity would provide a potent platform for testing hypotheses on healthy and diseased brain function in small rodents.

Brain imaging with PA in humans is currently hampered by strong scattering and absorption due to the skull. Although technically feasible, PA imaging in infants would, of course, have to pass rigorous safety assessments and prove significantly superior to US diagnosis before being implemented in clinics. PA imaging of peripheral nerve lesions in the diagnosis of diabetic neuropathy may thus more easily find its way to the clinic. Furthermore, label-free PA microscopy could accelerate safe diagnosis of the margin of resection during brain surgery. There are currently intense efforts to miniaturize endoscopic PA imaging probes for gastrointestinal indications (Ali et al., 2021) that can likely be extended to lumbar puncture or needle-based biopsies. Image-assistance could thus be provided to these procedures, which may also be augmented by spectroscopy or FDA-approved imaging agents such as ICG.

The future development of PA imaging in neuroscience depends as much on the technological development and fabrication of robust brain imaging platforms as it does on the bioengineering of reliable reporters of brain activity. Thus, close collaboration between biomedical engineers, chemists, materials scientists, synthetic biologists, and data scientists is desirable to foster further innovations that will make PA neuroimaging an integral part of the laboratory and clinic.

## Author Contributions

All authors listed have made a substantial, direct and intellectual contribution to the work, and approved it for publication.

## Conflict of Interest

The authors declare that the research was conducted in the absence of any commercial or financial relationships that could be construed as a potential conflict of interest.
